# Assessing the Impact of Manure Application in Commercial Swine Farms on the Transmission of Antimicrobial Resistant *Salmonella* in the Environment

**DOI:** 10.1371/journal.pone.0164621

**Published:** 2016-10-18

**Authors:** Suchawan Pornsukarom, Siddhartha Thakur

**Affiliations:** Department of Population Health and Pathobiology, College of Veterinary Medicine, North Carolina State University, Raleigh, North Carolina, 27607, United States of America; Natural Environment Research Council, UNITED KINGDOM

## Abstract

Land application of swine manure in commercial hog farms is an integral part of their waste management system which recycles the nutrients back to the soil. However, manure application can lead to the dissemination of bacterial pathogens in the environment and pose a serious public health threat. The aim of this study was to determine the dissemination of antimicrobial resistant *Salmonella* in the environment due to manure application in commercial swine farms in North Carolina (n = 6) and Iowa (n = 7), two leading pork producing states in the US. We collected manure and soil samples twice on day 0 (before and after manure application) from four distinct plots of lands (5 soil samples/plot) located at 20 feet away from each other in the field. Subsequent soil samples were collected again on days 7, 14, 21 from the same plots. A total of 1,300 soil samples (NC = 600; IA = 700) and 130 manure samples (NC = 60; IA = 70) were collected and analyzed in this study. The overall *Salmonella* prevalence was 13.22% (189/1,430), represented by 10.69% and 38.46% prevalence in soil and manure, respectively. The prevalence in NC (25.45%) was significantly higher than in IA (2.73%) (*P*<0.001) and a consistent decrease in *Salmonella* prevalence was detected from Day 0-Day 21 in all the farms that tested positive. *Salmonella* serotypes detected in NC were not detected in IA, thereby highlighting serotype association based on manure storage and soil application method used in the two regions. Antimicrobial susceptibility testing was done by the broth microdilution method to a panel of 15 antimicrobial drugs. A high frequency of isolates (58.73%) were multidrug resistant (resistance to three or more class of antimicrobials) and the most frequent resistance was detected against streptomycin (88.36%), sulfisoxazole (67.2%), and tetracycline (57.67%). Genotypic characterization by pulse field gel electrophoresis revealed clonally related *Salmonella* in both manure and soil at multiple time points in the positive farms. Our study highlights the potential role of swine manure application in the dissemination and persistence of antimicrobial resistant *Salmonella* in the environment.

## Introduction

Every year in the US more than a billion tons of manure is generated by livestock, primarily cattle (83%) followed by swine (10%) and poultry (7%) operations [1–2]. To deal with a large amount of manure, producers stack it in piles or store it in lagoons, and apply it on agricultural land to recycle the nutrients. Before application, the manure is often treated with thermophilic composting to inactivate potential pathogens that may be present [3–4]. However, there are concerns related to the dissemination of pathogenic bacteria from manure-amended soil which can contaminate water, food animals, and crops. Previous studies have reported the dissemination and persistence of infectious pathogens, antimicrobial residues, and antimicrobial resistance genes on agricultural field following manure application that subsequently enter the human food chain to become a public health hazard [5–11]. Several studies have reviewed the persistence of *Salmonella* in inoculated soil under various laboratory conditions over extended periods of time [12–14]. A study from Sub-Saharan Africa recovered *S*. Typhimurium six weeks after application of low-density inoculated manure and 14 weeks after application with high-density *Salmonella*-inoculated manure in a tropical climate [15]. *Salmonella* Typhimurium has been shown to persist in soil 180 days after application of cattle slurry in Sweden [16].

Manure generated in swine operations is usually collected, stored and treated in anaerobic lagoons or manure pits before being applied to the land as crops fertilizer [17–18]. The type of waste manage system used on each farm depends on multiple factors including the type of animal housing, manure handling during storage and land application, and geographic location. The pit systems are commonly used in the north-central region where the manure can be recycled back to cropland and the temperature is too cold for maintaining a lagoon [19]. The conventional approach is the pit-storage system which is located under the building and allows slurry to be stored for 120–180 days before being applied to the field. The pit-recharge system was developed to improve air quality inside livestock building [20–21]. The system, located under the ground, keeps most manure solids in suspension thereby making them easier to remove when the pit is drained. The anaerobic lagoon system was designed for anaerobic bacteria to decompose animal manure and convert manure to liquid that is easier for transportation and application [19, 22–23]. The important environmental concerns with anaerobic lagoons are odors, overflow, potential leakage, and over application of lagoon effluent [19]. The method used for manure application depends on the kind, volume and consistency of the manure, the hauling distance, costs, and existing equipment [24]. Liquid manure stored in lagoons is usually applied to the land through the use of irrigation sprinklers. This cheap method dilutes and forms manure into several small droplets or aerosols and has the potential of increased pathogen spreading, odor problems, and environmental concerns [25].

Studies aimed to determine the role of swine manure in the dissemination of *Salmonella* to the environment have either been conducted on a few commercial farms [26–27] or on experimental research stations [9, 28–29]. The other important concern relates to the potential dissemination of antimicrobial resistant (AMR) pathogens when swine manure is spread in the environment [30–31]. It is evident that there is a dearth of information on the potential movement of *Salmonella* from swine manure to other environmental niches in commercial swine farms. It is important to highlight that most of the studies estimating the dissemination of pathogens in the environment from animal manure are conducted on experimental research stations with spiked manure samples. To address this knowledge gap, we conducted a study in two leading pork producing states (North Carolina and Iowa) in the US to determine whether spreading manure in the environment leads to the dissemination of AMR *Salmonella*. We also compared the waste management system in the two states and its impact on *Salmonella* prevalence, serotype distribution, AMR-patterns, and pulsed-field gel electrophoresis (PFGE) profiles.

## Materials and Methods

### Farm distribution and waste management system

The sampling was conducted on a total of 13 commercial swine farms, including six sites in North Carolina (NC) and seven sites in Iowa (IA). Access to swine farms was approved by either the swine veterinarian or the farm owner. No samples were collected from vertebrate animals and the field studies did not involve an endangered or protected species. Protocols were discussed with the concerned authorities before proceeding onto the farm premises for sample collection and processing. In the seven IA farms that were sampled, the waste management system involved the use of a deep pit slurry system to store and treat swine manure. This is the preferred method of waste management system in swine farms in IA. Farms in Iowa store undiluted manure in pits and transfer the slurry to the fields by injection method. Soil injectors place liquid slurry into the soil at approximately 5–10 cm depth and cover by soil after the application. In NC, the farms sampled used an anaerobic lagoon system which is widely used in the state. Farms in North Carolina have wells on their property and use a flush system for dilution and manure removal from the housing to the anaerobic lagoons where they are stored in aerated ponds. Aerosolized lagoon waste is reduced into smaller particulate droplets and sprayed to the agricultural field using an irrigation sprinkler. The typical rate of manure application ranges from 4.2–4.7 liters/m^2^ for the injection method to 4.2–6.0 liters/m^2^ for the sprinkler approach [32]. In general, four slurry application methods are available, including the conventional method of broadcast spreading (splash plate), surface banding with trailing-hose (band spreading), trailing shoes, and injection [25, 33]. Injection method creates a furrow into the ground and fills manure within soil. This method is efficient in ammonium utilization bypassing infiltration, less air exposure of slurry, and environment-friendly, however, has a high energy-demand and specific soil requirements [25, 33].

### Sample collection

In both the states we visited each farm multiple times: day 0 (before and after manure application), day 7, day 14, and day 21 to study the potential dissemination and persistence of *Salmonella* in the soil following manure deposition. The soil samples were collected following the approach described previously with a few modifications [27]. A total of 1,430 samples were collected in the study, including 1,300 soil (NC: 600; IA: 700) and 130 manure samples (NC: 60; IA: 70). Manure samples (n = 10; 25 ml) were collected from the top 30 cm of lagoons or pits using 120-ml sterile containers during the first visit on each farm. Soil samples were collected twice on day 0 (before and after manure application) from four different plots within 0.4 hectare (4,000 m^2^) size of land (80 X 50 m). The farms in this study applied manure between 8–11 am in the morning. Before sample collection, 4 plots (1 m^2^ each) were identified at 20 feet apart from each other in a straight line and directly in line of the manure applicator. The plots were marked by flags for identification during subsequent sampling periods. We collected five soil samples (25 cm deep) weighing 100 gm each from every plot including the four corners and the middle of the plot. After 1–2 hours of manure application, soil samples were collected again from the same place in the plots (n = 20) on day 0. Overall, we collected a total 40 soil samples on day 0. This was followed by sequential visits on day 7, 14, and 21 to collect soil samples (n = 20) from the same spots. Samples originating in IA were shipped overnight at 4°C to NC and processed immediately in the laboratory.

### *Salmonella* isolation and confirmation

All the 1,430 samples were processed in NC for *Salmonella* isolation using standard methods described previously [34–36]. Briefly, 90 ml of buffered peptone water (BPW) (Difco, Becton-Dickinson, USA) was added into a Whirl-Pak bag containing 10 g of soil or 10 ml of manure sample and mixed thoroughly to be incubated at 37°C for 24 h. After pre-enrichment, a total of 100 μl of BPW suspension was transferred into 9.9 ml of Rappaport-Vassiliadis (RV) enrichment broth (Difco, Becton-Dickinson, USA) and incubated at 42°C for 24 h. A 10-μl loopful of enriched RV suspension was plated onto xylose lactose tergitol (XLT4) agar (Criterion, Hardy Diagnostics, USA) and incubated at 37°C overnight. A single black-colored colony from XLT4 plate was selected and inoculated by streaking and stabbing into triple sugar iron (TSI) and lysine iron agar (LIA) slants (Difco, Becton-Dickinson, USA) for biochemical testing. The presumptive *Salmonella* isolates that tested positive on TSI and LIA biochemical testing were confirmed by amplification of a targeted *Salmonella*-specific invasive (*inv*A) gene by PCR [37]. The isolates confirmed as *Salmonella* were labeled and stored in Brucella broth (Difco, Becton-Dickinson, USA) at -80°C until further characterization.

### *Salmonella* serotyping

The Kauffman-White scheme was applied for *Salmonella* serotyping. All *Salmonella* isolates (n = 189) were cultured overnight at 37°C on Luria-Bertani (LB) agar (Criterion, Hardy Diagnostics, USA) and sent to the National Veterinary Services Laboratories (NVSL) at Ames, Iowa for serotyping.

### Antimicrobial susceptibility testing

The broth microdilution method was used to determine the minimum inhibitory concentration (MIC) and antimicrobial resistance (AMR) profile of all confirmed *Salmonella* isolates recovered from soil and manure. This assay was carried out using Sensititre^®^ gram-negative CMV3AGNF plate (Trek Diagnostic Systems, Cleveland, OH, USA). The panel of 15 antimicrobials, abbreviation and respective concentration ranges, included are: amoxicillin/clavulanic acid (AUG2; 1/0.5-32/16 μg/ml), ampicilin (AMP; 1–32 μg/ml), azithromycin (AZI; 0.12–16 μg/ml), cefoxitin (FOX; 0.5–32 μg/ml), ceftiofur (XNL; 0.12–8 μg/ml), ceftriaxone (AXO; 0.25–64 μg/ml), chloramphenicol (CHL; 2–32 μg/ml), ciprofloxacin (CIP; 0.015–4 μg/ml), gentamicin (GEN; 0.25–16 μg/ml), kanamycin (KAN; 8–64 μg/ml), nalidixic acid (NAL; 0.5–32 μg/ml), streptomycin (STR; 32–64 μg/ml), sulfisoxazole (FIS; 16–256 μg/ml), trimetroprim/sulfamethoxazole (SXT; 0.12/2.38-4/76 μg/ml), and tetracycline (TET; 4–32 μg/ml). The MICs were determined and breakpoints were interpreted based on the Clinical and Laboratory Standards Institute standards (CLSI) for broth microdilution [38] and National Antimicrobial Resistance Monitoring System (NARMS) [39]. *E*. *coli* ATCC 25922 was used as reference strain to measure sensitivity. The isolates interpreted as intermediate level were categorized into susceptible to avoid overestimation of resistance. The isolates with resistance to three or more classes of antimicrobials were classified as multidrug resistance (MDR).

### Pulse field gel electrophoresis (PFGE) analysis

*Salmonella* isolates from soil (n = 139) and manure (n = 50) recovered from different commercial swine farms in NC and IA were genotyped using PFGE following the PulseNet protocol from the Centers for Disease Control and Prevention (CDC) [40]. In brief, *Salmonella* isolates were grown on LB agar plates at 37°C for 14–18 h. Cell suspension buffer (CSB; 100 mM Tris and 100 mM EDTA, pH 8.0) was used to suspend and adjust the bacterial concentration to an optical density (OD) of 0.48–0.52 using a Dade MicroScan Turbidity meter. TE buffer (10 mM Tris and 1 mM EDTA, pH 8.0), cell lysis buffer (CLB; 50 mM Tris, 50 mM EDTA: pH8 and 1% sarcosyl) and proteinase K (20 mg/ml) were used to prepare agarose embedded cells. After the bacterial cells were lysed, intact genomic DNA was digested with 50 U of *Xba*I restriction enzyme (New England Biolabs, Ipswich, MA, USA) at 37°C for 2 h. The PulseNet universal strain *Salmonella enterica* serovar Braenderup H9812 was used as a molecular standard marker. The DNA fragments were separated by CHEF-DR^®^III Pulsed-Field Electrophoresis System (Bio-Rad Laboratories, Hercules, CA, USA) at 14°C for 18 h. BioNumerics software version 6.1 (Applied Maths, Kortrijk, Belgium) was used to analyzed the PFGE images. The clonal relatedness was determined using the Dice coefficient similarity index and unweighted-pair group average (UPGMA) cluster analysis with 2.0% optimization and 2.0% tolerance banding pattern. PFGE fingerprint patterns with a similarity index >90% were clustered within the same genotypic group.

### Statistical analysis

Pearson’s Chi-square analysis was performed to test difference in *Salmonella* prevalence between sample types (manure and soil), manure storage system (lagoon or pit), and state of origin (NC and IA). A value of *P* < 0.05 was considered statistically significant finding. Strength of association between serotype and AMR pattern was determined using the odds ratio (OR) with a 95% confidence interval. All data analysis was carried out using R version 3.1.2 (R foundation for statistical computing, Vienna, Austria).

## Results

### *Salmonella* prevalence in swine farms environment in NC and IA

**Fig 1:**
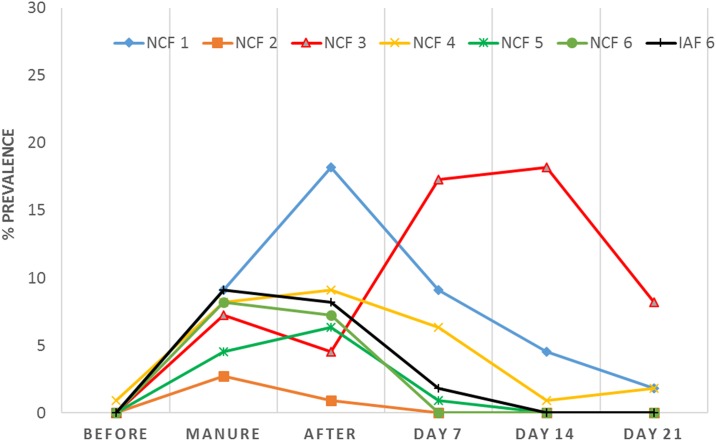
*Salmonella* prevalence among North Carolina samples (NCF 1-NCF 6) and Iowa samples (IAF 6) at different time points.

A significantly higher prevalence of *Salmonella* was detected in NC (168/660, 25.45%) than IA (21/770, 2.73%) for a total of 189 *Salmonella* isolates in the study (*P*<0.0001). We isolated *Salmonella* from all the six farms tested in NC while only a single farm in IA (IAF 6) tested positive (Fig 1). *Salmonella* prevalence in manure (50/130; 38.46%) samples was significantly higher than in soil (139/1,300; 10.69%) (*P* < 0.0001). Of the 60 manure samples from NC, 40 (66.67%) were positive for *Salmonella*, while only 10 out of 70 manure samples (14.29%) from IA were positive (*P* < 0.0001). A total of 128 (21.33%) out of 600 soil samples from NC and 11 (1.57%) out of 700 IA soil samples tested positive for *Salmonella*, respectively (*P* < 0.0001).

The prevalence of *Salmonella* in farm environment at different time points following land application were highest on day 0, especially from the manure collected directly from the lagoon/pit and the soil samples collected immediately after manure application. *Salmonella* prevalence tended to decrease in subsequent weeks, except in NCF 3 where the prevalence increased on future samplings done on days 7, 14 and 21 (Fig 1). Rarely *Salmonella* was detected on land before manure application, except for a single soil sample collected from NCF 4. We detected variation in serotype distribution between farms. For example, in NCF 1, *Salmonella* serotypes Altona, Mbandaka, Muenster, Uganda, and Worthington were recovered in manure lagoon as well as and manure enriched soil on the first visit but none of these serotypes were recovered from soil before manure application. The five serotypes persisted in the soil samples until day 7. After two weeks, we detected only serotypes *S*. Altona and *S*. Muenster in the soil samples from NCF 1 and finally only *S*. Altona was recovered on the last visit (day 21). Similarly, *Salmonella* Derby and Ohio were recovered from manure samples in NCF 5. We did not recover any *Salmonella* in the field before manure application on this farm. After two hour of land application, we detected *S*. Derby and *S*. Ohio from the same soil samples. However, they were not recovered in the next sequential visits on days 7, 14 and 21.

### Identification and distribution of *Salmonella* serotypes

The 189 *Salmonella* isolates were represented by 18 different serotypes (Table 1). The serotypes detected in one state were not reported from the other. Three *Salmonella* serotypes were identified in IA, including *Salmonella* Anatum (6.88%), *S*. Litchfield (3.70%), and *S*. Infantis (0.53%). We observed a wider distribution of *Salmonella* serotypes in NC predominantly represented by *S*. Typhimurium var5- (22.22%), *S*. Senftenberg (14.81%), *S*. Rissen (8.99%) and *S*. Muenster (8.47%). Majority of the NC swine lagoon samples were represented by *S*. Rissen. There was a wide distribution of serotypes detected within each NC farm and some serotypes were detected in more than one farm including Worthington, Johannesburg, Derby, Rissen, and Typhimurium var5- (Table 1). We observed persistence of specific *Salmonella* serotypes throughout a farm in all the samples collected after manure application. This was seen in case of *S*. Altona and *S*. Muenster serotypes in samplings conducted on NCF 1, while *S*. Typhimurium var5- and *S*. Johannesburg were prevalent in samples collected from NCF 3. In contrast, *S*. Senftenberg was isolated from NCF 4 throughout the sampling period at all stages, including from soil before manure application.

**Table 1:** Distribution of *Salmonella* serotypes by farms at different time points following manure application.

Farms	Day 0 (n; %)^c^			Day 7^b^ (n; %)^c^	Day 14^b^ (n; %)^c^	Day 21^b^ (n; %)^c^
(n = 189)	Manure^a^	Before^b^	After^b^			
**NCF 1**	Altona (1; 2.13%)		Altona (5; 10.64%)	Altona (4; 8.51%)	Altona (2; 4.26%)	Altona (2; 4.26%)
(n = 47)	Mbandaka (1; 2.13%)		Mbandaka (1; 2.13%)	Mbandaka (1; 2.13%)		
	Muenster (5; 10.64%)		Muenster (5; 10.64%)	Muenster (3; 6.38%)	Muenster (3; 6.38%)	
	Uganda (2; 4.26%)		Uganda (1; 2.13%)	Uganda (1; 2.13%)		
	Worthington (1; 2.13%)		Worthington (8; 17.02%)	Worthington (1; 2.13%)		
**NCF 2**	Derby (2; 50%)					
(n = 4)	Rough_O:z10:e,n,z15 (1; 25%)					
			Johannesburg (1; 25%)			
**NCF 3**				Derby (1; 1.69%)	Derby (3; 5.08%)	Derby (1; 1.69%)
(n = 59)	Johannesburg (4; 6.78%)			Johannesburg (2; 3.39%)	Johannesburg (1; 1.69%)	
	Rissen (1; 1.69%)					
	Typhimurium var5- (2; 3.39%)			Typhimurium var5- (15; 25.42%)	Typhimurium var5- (16; 27.12%)	Typhimurium var5- (8; 13.56%)
			Worthington (5; 8.47%)			
**NCF 4**				6,7:-:e,n,z15 (1; 3.33%)		
(n = 30)						Mbandaka (1; 3.33%)
	Senftenberg (9; 30%)	Senftenberg (1; 3.33%)	Senftenberg (10; 33.33%)	Senftenberg (6; 20%)	Senftenberg (1; 3.33%)	Senftenberg (1; 3.33%)
**NCF 5**	Derby (1; 9.09%)		Derby (1; 9.09%)			
(n = 11)	Ohio (3; 27.27%)		Ohio (4; 36.36%)			
				Ouakam (1; 9.09%)		
	Typhimurium var5- (1; 9.09%)					
**NCF 6**			4,12:i:- (1; 5.88%)			
(n = 17)	Rissen (9; 52.94%)		Rissen (7; 41.18%)			
**IAF 6**	Anatum (10; 47.62%)		Anatum (2; 9.52%)	Anatum (1; 4.76%)		
(n = 21)			Infantis (1; 4.76%)			
			Litchfield (6; 28.57%)	Litchfield (1; 4.76%)		

^a^ Manure (NCF 1–6) in North Carolina is stored in lagoons while in Iowa (IAF 6), it is stored in the form of slurry in pits.

^b^ Soil samples

^c^ The percentage was calculated within each commercial swine farm.

### Antimicrobial resistance profile of *Salmonella*

A total of 189 *Salmonella* isolates (NC = 168, IA = 21) were tested for AST using Sensititre^®^ containing a panel of 15 antimicrobial drugs. A squashtogram was created to represent the MIC distribution and AMR profile of *Salmonella* isolated in NC and IA swine farms (Table 2). *Salmonella* isolates exhibited highest frequency of resistance to STR (88.36%) followed by FIS (67.2%) and TET (57.67%). A large proportion of the AMR *Salmonella* isolates were MDR (111/189; 58.73%), including a significantly higher number in NC (63.1%) than IA (23.81%) (*P* = 0.001). Only 8.47% of total isolates were pan-susceptible which was observed predominantly in serotype Anatum isolated from IA manure samples. The highest frequency of resistance in NC was exhibited to STR (89.29%), FIS (73.21%), and TET (63.69%) while in IA, the *Salmonella* isolates were predominantly resistant to STR (80.95%), AXO and XNL (23.81%), and FIS and FOX (19.05%). In addition, we observed that NC isolates also exhibited resistance to other aminoglycosides including KAN (47.02%), and GEN (17.26%). None of the *Salmonella* isolates were resistant to AZI, CIP, and NAL. The most frequent AMR patterns, associated serotypes, and their distributions are categorized in Table 3. AMP FIS KAN STR (n = 19) was the most common MDR pattern that was identified in NC from both lagoon and soil samples and was found to be significantly associated with *S*. Typhimurium var5- (*P*<0.0001; OR = 120.61). Another major MDR pattern associated with *S*. Typhimurium var5- was FIS KAN STR (n = 17) (*P*<0.0001; OR = ∞). This later pattern was only found in soil sample from NC. *S*. Senftenberg (n = 28) and *S*. Worthington (n = 15) were the frequent serotypes isolated from NC and were also associated with MDR patterns (*P*<0.0001: OR _Senftenberg_ = 215.28, OR_Worthington_ = 15.9). The most common serotype found in NC lagoon (*S*. Rissen; n = 17) was associated with the STR TET pattern.

**Table 2:** Comparison of resistance and MIC distribution for *Salmonella* isolated in North Carolina and Iowa (NC = 168; IA = 21).

AM^a^	Origin	%R^b^	[95% CI]	Distribution of MICs in μg/mL (%)															
				0.015	0.03	0.06	0.125	0.25	0.5	1	2	4	8	16	32	64	128	256	512
**AMP**	NC	25.60	[19.0–32.2]						51.8	1.8	13.7	3.6	1.2	2.4	2.4	23.2			
	IA	14.29	[-0.7–29.3]						33.3	0.0	42.9	4.8	0.0	4.8	0.0	14.3			
**AUG2**	NC	7.14	[3.3–11.0]						61.3	0.0	40.7	1.2	1.2	24.4	4.2	3.6			
	IA	14.29	[-0.7–29.3]						66.7	0.0	14.3	0.0	0.0	4.8	4.8	9.5			
**AXO**	NC	5.36	[2.0–8.8]					91.1	2.4	0.0	1.2	0.0	1.2	3.6	0.6	0.0			
	IA	23.81	[5.6–42.0]					76.2	0.0	0.0	0.0	0.0	0.0	14.3	4.7	4.7			
**AZI**	NC	0	[0.0]				0.0	0.0	0.0	3.0	18.5	57.1	20.2	1.2					
	IA	0	[0.0]				0.0	0.0	0.0	0.0	0.0	42.9	57.1	0.0					
**CHL**	NC	1.19	[-0.5–2.8]								0.0	17.9	79.8	1.2	1.2				
	IA	0	[0.0]								0.0	9.5	85.7	4.8	0.0				
**CIP**	NC	0	[0.0]	79.2	19.0	1.2	0.0	0.6	0.0	0.0	0.0	0.0							
	IA	0	[0.0]	47.6	47.6	4.8	0.0	0.0	0.0	0.0	0.0	0.0							
**FIS**	NC	73.21	[66.5–79.9]											3.0	2.4	14.9	6.0	0.6	73.2
	IA	19.05	[2.3–35.9]											0.0	9.5	23.8	14.3	33.3	19.0
**FOX**	NC	6.55	[2.8–10.3]						0.0	0.6	18.5	66.7	5.4	2.4	4.2	2.4			
	IA	19.05	[2.3–35.9]						0.0	0.0	19.0	52.4	4.8	4.8	9.5	9.5			
**GEN**	NC	17.26	[11.6–23.0]					1.8	54.8	25.0	1.2	0.0	0.0	1.2	16.1				
	IA	0	[0.0]					28.6	42.9	28.6	0.0	0.0	0.0	0.0					
**KAN**	NC	47.02	[39.5–54.6]									51.8	0.0	0.0	1.2	3.6	43.5		
	IA	14.29	[-0.7–29.3]									81.0	0.0	0.0	4.8	0.0	14.3		
**NAL**	NC	0	[0.0]						0.0	0.0	42.3	56.0	1.2	0.6	0.0				
	IA	0	[0.0]						0.0	9.5	0.0	85.7	4.8	0.0	0.0				
**STR**	NC	89.29	[84.6–94.0]												10.7	13.1	76.2		
	IA	80.95	[64.2–97.8]												19.0	0.0	81.0		
**SXT**	NC	5.36	[2.0–8.8]			71.4	1.2	14.3	7.7	0.0	0.0	0.0	5.4						
	IA	4.76	[-4.4–13.9]			95.2	0.0	0.0	0.0	0.0	0.0	0.0	4.8						
**XNL**	NC	5.36	[2.0–8.8]				0.0	0.0	20.2	72.6	0.0	1.8	5.4						
	IA	23.81	[3.04–13.2]				0.0	0.0	0.0	71.4	4.8	0.0	23.8						
**TET**	NC	63.69	[56.4–71.0]								19.6	16.7	0.0	1.2	1.2	61.3			
	IA	9.52	[35.8–54.3]								90.5	0.0	0.0	4.8	0.0	4.8			

The vertical bars indicate the breakpoints for resistance.

^a^ amoxicillin/clavulanic acid (AUG2; 1/0.5-32/16 μg/ml), ampicilin (AMP; 1–32 μg/ml), azithromycin (AZI; 0.12–16 μg/ml), cefoxitin (FOX; 0.5–32 μg/ml), ceftiofur (XNL; 0.12–8 μg/ml), ceftriaxone (AXO; 0.25–64 μg/ml), chloramphenicol (CHL; 2–32 μg/ml), ciprofloxacin (CIP; 0.015–4 μg/ml), gentamicin (GEN; 0.25–16 μg/ml), kanamycin (KAN; 8–64 μg/ml), nalidixic acid (NAL; 0.5–32 μg/ml), streptomycin (STR; 32–64 μg/ml), sulfisoxazole (FIS; 16–256 μg/ml), trimetroprim/sulfamethoxazole (SXT; 0.12/2.38-4/76 μg/ml), and tetracycline (TET; 4–32 μg/ml)

^b^ Percent resistant isolates to each antimicrobial in a state.

**Table 3:** Distribution of *Salmonella* serotypes associated with predominant R-patterns.

*Salmonella* serotypes (n)	Predominant patterns^a^ (n)	Manure n(%)^b^	Soil n(%)^b^
Typhimurium var5-^1^ (42)	AMP FIS KAN STR (19)	2 (10.53)	17 (89.47)
	FIS KAN STR (17)	0	17 (100)
	AMP AUG2 FIS KAN STR (2)	0	2 (100)
	FIS KAN STR TET (2)	0	2 (100)
	AMP CHL FIS KAN STR TET (1)	1 (100)	0
	AMP FIS STR (1)	0	1 (100)
Senftenberg^1^ (28)	FIS STR TET (25)	7 (28.0)	18 (72.0)
	AMP FIS STR TET (2)	2 (100)	0
	AMP AUG2 FIS FOX KAN STR TET (1)	0	1 (100)
Worthington^1^ (15)	FIS GEN KAN STR TET (9)	0	9 (100)
	STR TET (5)	0	5 (100)
	FIS GEN KAN STR SXT TET (1)	0	1 (100)
Rissen^1^ (17)	STR TET (15)	9 (60)	6 (40)
	AMP CHL FIS STR SXT TET (1)	1 (100)	0
	AMP STR TET (1)	0	1 (100)
Anatum^2^ (13)	Pan-susceptible (3)	3 (100)	0
	STR (3)	1 (33.33)	2 (66.67)
	AMP AUG2 AXO FOX KAN STR XNL (2)	2 (100)	0
	FIS STR (2)	1 (50)	1 (50)
	AMP AXO FIS STR XNL (1)	1 (100)	0
	AUG2 AXO FOX KAN STR XNL (1)	1 (100)	0
	AXO FOX XNL (1)	1 (100)	0

^a^ ampicillin (AMP), amoxicillin/clavulanic acid (AUG2), cefoxitin (FOX), ceftiofur (XNL), ceftriaxone (AXO), chloramphenicol (CHL), kanamycin (KAN), streptomycin (STR), sulfisoxazole (FIS), trimetroprim/sulfamethoxazole (SXT), and tetracycline (TET)

^b^ number of isolates (percent resistant to a specific R-pattern)

^1^ *Salmonella* serotypes isolated from commercial swine farms in North Carolina.

^2^ *Salmonella* serotype isolated from commercial swine farm in Iowa.

### Pulse field gel electrophoresis (PFGE)

**Fig 2:**
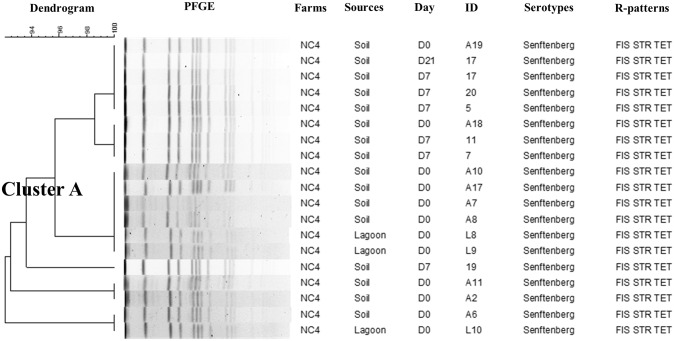
Phylogenetic analysis representing PFGE-*Xba*I with antimicrobial resistant patterns of *Salmonella* Senftenberg from NCF 4 at 90% cut-off genotypic similarity (Cluster A).

**Fig 3:**
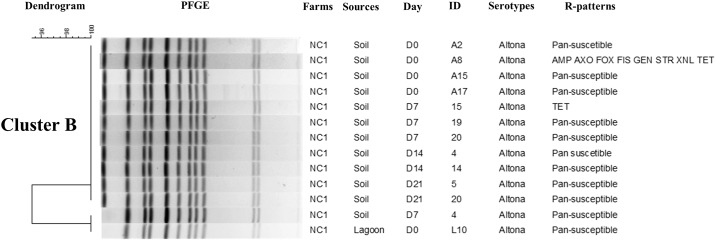
Phylogenetic analysis representing PFGE-*Xba*I with antimicrobial resistant patterns of *Salmonella* Altona from NCF 1 at 90% cut-off genotypic similarity (Cluster B).

**Fig 4:**
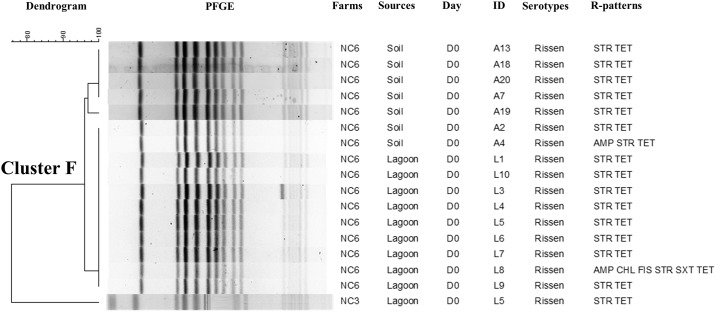
Phylogenetic analysis representing PFGE-*Xba*I with antimicrobial resistant patterns of *Salmonella* Rissen from NCF 3&6 at 90% cut-off genotypic similarity (Cluster F).

Genotypic characterization using PFGE with *Xba*I restriction enzyme generated on an average 10 to 18 DNA bands and distributed the *Salmonella* isolates (n = 189) into seven major clusters represented by NC (six clusters) and IA (one cluster) (S1 Fig). Each individual major cluster was represented by *Salmonella* isolates belonging to the same serotype and were related to farm of origin. Distinct serotype distributions were detected in the different NC farms as exhibited in the three separate dendrograms that were created (Figs 2–4). *S*. Senftenberg (Cluster A) (Fig 2) isolated from multiple sampling points in NCF 4, including manure and day0 (after manure application), days7, 14 and 21 soil samples, had 100% similar PFGE profiles. In addition, all *S*. Senftenberg isolates in this cluster were MDR and shared the same R-pattern (FIS STR TET) highlighting the dissemination and persistence of this serotype after manure application based on phenotypic and genotypic characterization. Similarly, we detected genotypic similar *S*. Altona (Cluster B) isolated from NCF 1 soil at different time points (day0, day7, day14, and day21) (Fig 3). A single isolate from lagoon was grouped in this cluster and had similar PFGE pattern with another *S*. Altona isolated from soil on day7. The isolates in this cluster were predominantly pan-susceptible. Finally, *S*. Rissen (Cluster F) from NCF 6 on day 0 from lagoon and soil after manure application were genotypically identical (Fig 4). We detected two clusters that were composed of *Salmonella* isolated only from soil and not from manure (S1 Fig). This includes Cluster D (serotype Litchfield; n = 5) and E (*S*. Typhimurium var5-; n = 23). We used a 90% cut-off genotypic similarity to create the different clusters for our analysis. Using a less stringent cut-off value would have created bigger clusters, however we used a conservative approach. Seventy seven isolates did not cluster in any specific group and were represented as singletons.

## Discussion

To date, no comprehensive research has been conducted on commercial swine farms to study the dissemination and persistence of AMR *Salmonella* from swine manure systems to soil environment after land application. The main objective of this study was to determine whether swine manure application in the farm environment leads to dissemination of *Salmonella*. In addition, we wanted to determine the impact of geographic location and the distinct waste management systems in the two states on *Salmonella* prevalence. Swine farms sampled in NC used a lagoon system for swine manure disposal while the farms covered in IA typically used a deep-pit storage system to store manure before applied on agricultural lands. In our study, *Salmonella* prevalence was significantly higher in manure samples than in soil samples (*P* < 0.0001). We observed a decrease in prevalence of *Salmonella* at different time points of sampling date (from day0 to day21), except in NCF 3 where the prevalence increased between day7-day14 after manure application. Based on our records, this farm had experienced a heavy rainfall event before sampling on day 14. Studies have explored the association between rainfall and microbial contamination where heavy rainfall events before dry spells have shown to assist in pathogen dissemination [41–42]. We observed that *Salmonella* can persist on land at least 3 weeks after swine manure application. Factors contributing to the survival of *Salmonella* in soil include temperature, moisture, soil type, plants, UV light, and soil organisms [43]. In contrast to our study, *Salmonella* was rarely isolated in the soil samples before manure application in both states. We isolated *Salmonella* from a single farm in IA while all six farms in NC tested positive. *Salmonella* prevalence was found to be dependent on the swine manure storage system (lagoon or pit). Further, the subsequent dissemination and persistence in the environment was found to be dependent on the manure application method (spraying or injection) being employed on the farm. Once disseminated, *Salmonella* can persist in the environment for a significant amount of time depending on the geographic location and weather prevalent in the region. *Salmonella spp*. has been reported to survive in manure-amended soils from 2–3 until 332 days [13, 43–45]. Even in the absence of active fertilization, *Salmonella* has been isolated ubiquitously in environmental soil samples collected from agricultural and recreational areas [46–47]. It is quite possible that the soil characteristics and weather conditions may have a direct impact on pathogen survival than in the manner the manure was applied.

Multiple *Salmonella* serotypes were identified in our study and none of the serotypes detected in one state were reported from the other. The most common serotypes detected in NC farms environment were Typhimurium var5-, Senftenberg, and Rissen. Previous study in NC reported that the predominant serotypes isolated from swine farms were Typhimurium followed by Infantis, Derby, and Anatum [34]. In contrast, *S*. Anatum was not identified in NC, but was the predominant serovar in IA pit and soil samples. Abley et al. [48] reported that the top three *Salmonella* serotypes over the leading swine producing states (Iowa, North Carolina, and Minnesota) were Typhimurium (42%), Derby (25%) and Adelaide (5%). These reports are in agreement with the CDC annual surveillance data which reports the most frequent *Salmonella* serotypes from porcine source are *S*. Typhimurium, *S*. Derby, *S*. Agona, *S*. Infantis, and *S*. Heidelberg [49]. We observed that *S*. Rissen was one of the most common serotypes in NC farms especially in the manure samples from lagoon. This serotype was not well-known until the outbreak in California 2008–2009 which resulted from the consumption of white ground pepper imported from Asia [49–51]. *S*. Rissen is the most frequent and dominant serotype presented in south-east Asian countries especially in swine herds and retail pork and is reported to be MDR [52–55]. In contrast, *S*. Rissen was identified for the first time in swine herds and environment in NC in the year 2009 [34].

*Salmonella* isolates from our study in both NC and IA were resistant to various classes of antimicrobials including streptomycin (88.36%), sulfisoxazole (67.2%), tetracycline (57.67%), kanamycin (43.39%) while 58.73% were MDR. These results are in accordance with previous studies in swine production where tetracycline resistant *Salmonella* were reported in the highest frequency followed by streptomycin and sulfisoxazole [34, 48, 56]. Heuer et al. [57] documented that the antimicrobial compounds of sulfamethazine, tetracycline, chlortetracycline and tylosin used in farms were recovered from spread manure to agricultural soils. This evidence supports the association between agricultural antimicrobial use and its resistance [31]. The excessive or inappropriate applications of antimicrobials in food animals described as therapeutic, prophylactic, and sub-therapeutic uses are considered to be the key of antimicrobial resistance problem [31]. Department of Agriculture [58] reported that approximately 88% of commercial swine farms in US used antimicrobials, frequently tetracycline or tylosin in their feed for disease prevention and growth promotion purposes. Most antimicrobial uses in farms need prescriptions from veterinarians, even though the particular treatment decisions are administrated by the farm workers [31]. The most common MDR-patterns in our study were FIS STR TET (16.4%), FIS GEN KAN STR TET (12.7%), and AMP FIS KAN STR (10.58%) which were significantly associated with serotypes Senftenberg, Worthington, and Typhimurium var5-, respectively. *S*. Typhimurium is common in swine production and mostly observed as MDR [34, 48, 59]. The high frequency of resistance to different antimicrobials in *Salmonella* isolates (91.53%) recovered in our study from manure and soil samples is concerning. This is especially true in our study since majority of the soil samples before manure application were negative for the pathogen. Therefore, based on our study results we state that swine manure application does leads to dissemination of AMR *Salmonella* to the farm environment. However, it is important to note that our study was conducted on limited number of commercial swine farms and has limited internal and external validity.

Genotypic characterization by PFGE distributed the 189 *Salmonella* isolates into seven major clusters based on serotype and has been reported previously [48, 52, 60]. The presence of clonal *Salmonella* isolates with identical phenotypic R-patterns suggests an epidemiological link between *Salmonella* recovered from manure and soil at different time points. The PFGE profiles (Figs 2–4 and S1) confirms the finding that *Salmonella* can be disseminated from manure use and persist in the environment at least 3 weeks after land application which is in accordance with serotype distribution in each farm at different time points (Table 1). Bech et al. [12] reported detecting *Salmonella* up to a month after application in loamy soil under cold and moist conditions. *S*. Typhimurium has been shown to persist in pig slurry applied to a Danish field up to 14 days [61] while *E*. *coli* has been detected on day 21 after manure amendment [27]. Studies have reported the presence of pathogens, AMR genes, and antimicrobial residues in lagoons and on lands after exposed to the swine manure [8, 10, 62–63]. According to the study results, we observed that *Salmonella* presents in swine manure, when spread on land, can persist for at least 21 days in soil (the longest period that could be detected in our study). However, the period of persistence varied among farms and states of origin. It is also important to highlight that not all clusters were represented by *Salmonella* isolates from soil and manure. Cluster D and E consisted of *Salmonella* serotypes that were isolated only from the soil. Clearly it is possible that these specific strains representing different *Salmonella* serotypes were already present in the soil even before manure application.

## Conclusion

Based on phenotypic and genotypic characterization, our study highlighted the potential dissemination of AMR *Salmonella* after swine manure application in the environment. *Salmonella* present in swine manure, after spread on land, was able to persist in soil for at least 21 days in three out of the seven farms that were positive for the pathogen. The persistence of *Salmonella* in manure amended soils can have important public health implications. The dissemination of AMR *Salmonella* was dependent on the geographic location of the farm, waste storage system and on the specific manure application approach employed by the farm management. We acknowledge that the soil characteristics and existing weather conditions may have an impact on *Salmonella* survivability. It will be important to conduct future comprehensive longitudinal and quantitative based study to study the dissemination of AMR pathogens from livestock manure application in the environment.

## Supporting Information

S1 Fig Phylogenetic analysis representing PFGE-*Xba*I with antimicrobial resistant patterns of *Salmonella* isolated from NC and IA commercial swine farms at 90% cut-off genotypic similarity (Cluster A-G). (PDF)
