# Supplementary material for: Assessing the Impact of Manure Application in Commercial Swine Farms on the Transmission of Antimicrobial Resistant Salmonella in the Environment
Source: PLoS One. 2016 Oct 18;11(10):e0164621. doi: 10.1371/journal.pone.0164621 (PMC5068702; doi:10.1371/journal.pone.0164621)
Supplement: S1 Fig — (PDF) [file pone.0164621.s001.pdf]

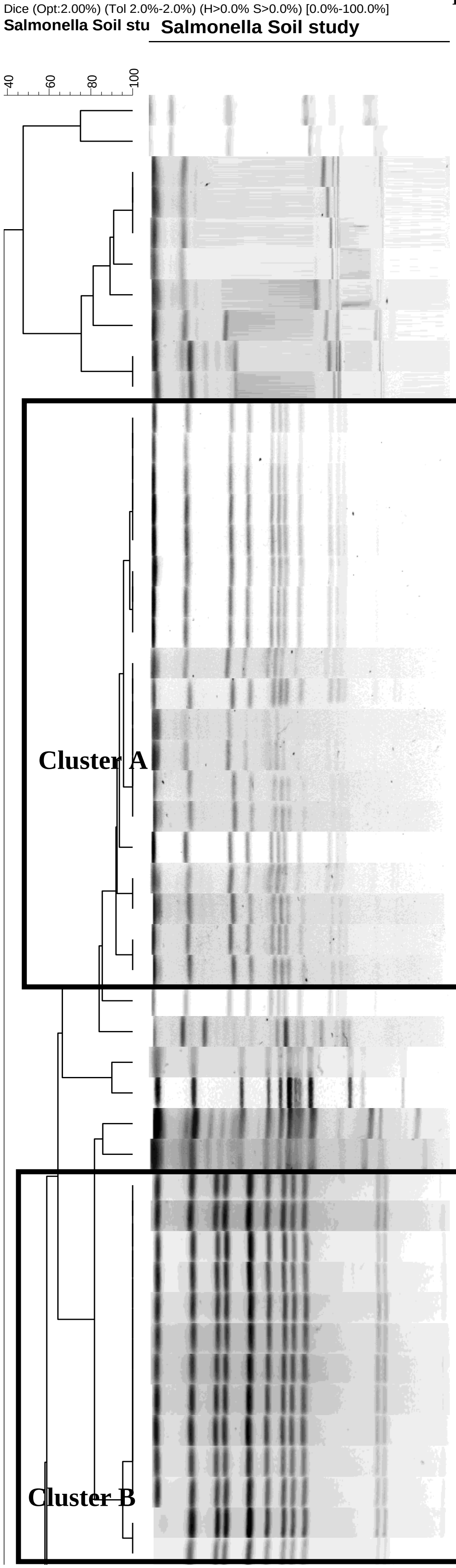

Farms	Sources	Day	ID	Serotypes	R-patterns
IA6	Soil	D0	A3	Litchfield	STR
IA6	Soil	D0	A5	Litchfield	FIS STR
NC4	Lagoon	D0	L3	Senftenberg	FIS STR TET
NC4	Lagoon	D0	L4	Senftenberg	FIS STR TET
NC4	Lagoon	D0	L5	Senftenberg	FIS STR TET
NC4	Lagoon	D0	L6	Senftenberg	AMP FIS STR TET
NC4	Lagoon	D0	L2	Senftenberg	FIS STR TET
NC4	Lagoon	D0	L1	Senftenberg	AMP FIS STR TET
NC3	Soil	D14	17	Derby	FIS KAN STR TET
NC3	Soil	D14	18	Typhimurium var5-	AMP FIS KAN STR
NC4	Soil	D0	A19	Senftenberg	FIS STR TET
NC4	Soil	D21	17	Senftenberg	FIS STR TET
NC4	Soil	D7	17	Senftenberg	FIS STR TET
NC4	Soil	D7	20	Senftenberg	FIS STR TET
NC4	Soil	D7	5	Senftenberg	FIS STR TET
NC4	Soil	D0	A18	Senftenberg	FIS STR TET
NC4	Soil	D7	11	Senftenberg	FIS STR TET
NC4	Soil	D7	7	Senftenberg	FIS STR TET
NC4	Soil	D0	A10	Senftenberg	FIS STR TET
NC4	Soil	D0	A17	Senftenberg	FIS STR TET
NC4	Soil	D0	A7	Senftenberg	FIS STR TET
NC4	Soil	D0	A8	Senftenberg	FIS STR TET
NC4	Lagoon	D0	L8	Senftenberg	FIS STR TET
NC4	Lagoon	D0	L9	Senftenberg	FIS STR TET
NC4	Soil	D7	19	Senftenberg	FIS STR TET
NC4	Soil	D0	A11	Senftenberg	FIS STR TET
NC4	Soil	D0	A2	Senftenberg	FIS STR TET
NC4	Soil	D0	A6	Senftenberg	FIS STR TET
NC4	Lagoon	D0	L10	Senftenberg	FIS STR TET
NC4	Soil	D0	A5	Senftenberg	FIS STR TET
NC4	Soil	D0	B11	Senftenberg	AMP AUG2 FOX FIS KAN STR TET
NC3	Soil	D21	1	Derby	FIS STR TET
NC3	Soil	D7	17	Derby	AMP AUG2 AXO FIS KAN STR XNL TE
NC3	Soil	D14	10	Derby	FIS STR TET
NC3	Soil	D14	5	Derby	FIS STR TET
NC1	Soil	D0	A2	Altona	Pan-suscetible
NC1	Soil	D0	A8	Altona	AMP AXO FOX FIS GEN STR XNL TET
NC1	Soil	D0	A15	Altona	Pan-susceptible
NC1	Soil	D0	A17	Altona	Pan-susceptible
NC1	Soil	D7	15	Altona	TET
NC1	Soil	D7	19	Altona	Pan-susceptible
NC1	Soil	D7	20	Altona	Pan-susceptible
NC1	Soil	D14	4	Altona	Pan suscetible
NC1	Soil	D14	14	Altona	Pan-susceptible
NC1	Soil	D21	5	Altona	Pan-susceptible
NC1	Soil	D21	20	Altona	Pan-susceptible
NC1	Soil	D7	4	Altona	Pan-susceptible
NC1	Lagoon	D0	L10	Altona	Pan-susceptible

Dendrogram PFGE

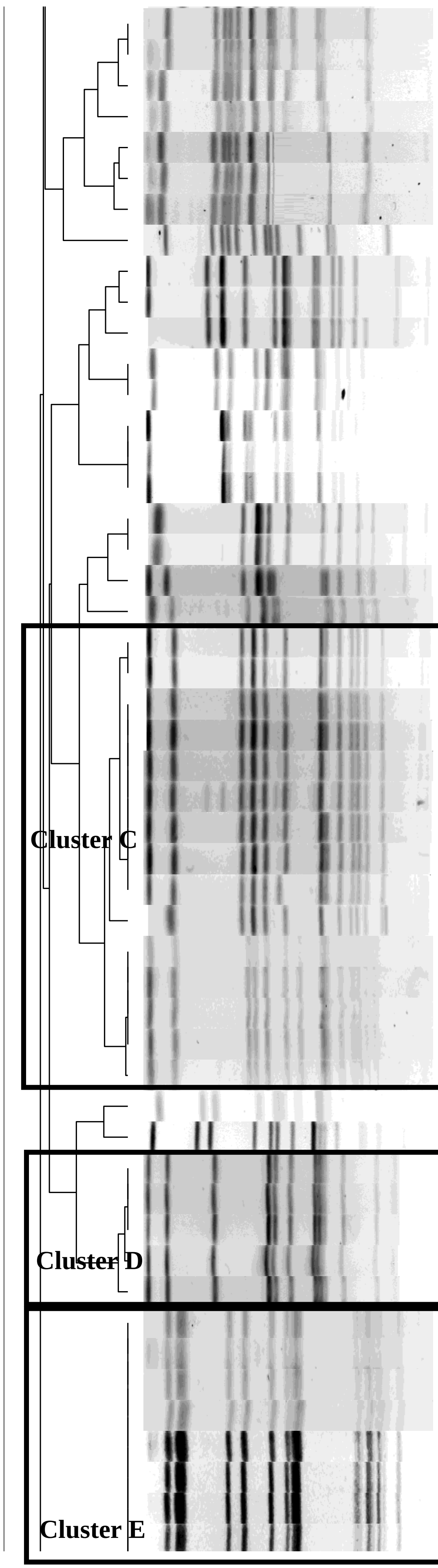

Farms	Sources	Day	ID	Serotypes	R-patterns
NC3	Soil	D7	6	Johannesburg	AUG2 AXO FOX FIS KAN STR XNL TET
NC3	Soil	D7	7	Johannesburg	FIS STR
NC3	Lagoon	D0	L1	Johannesburg	AMP AUG2 AXO FOX FIS KAN STR XNL
NC3	Lagoon	D0	L2	Johannesburg	AMP AUG2 AXO FOX FIS KAN STR XNL
NC3	Lagoon	D0	L7	Johannesburg	AMP AUG2 AXO FOX FIS KAN STR XNL
NC3	Soil	D14	20	Johannesburg	AMP FIS KAN STR
NC3	Lagoon	D0	L10	Johannesburg	AMP AUG2 AXO FOX FIS KAN STR XNL
NC2	Soil	D0	A2	Johannesburg	KAN STR SXT TET
NC1	Soil	D0	A19	Mbandaka	FIS SXT
NC1	Soil	D7	6	Mbandaka	FIS SXT
NC1	Lagoon	D0	L2	Mbandaka	FIS SXT
NC5	Soil	D0	A4	Derby	AMP STR TET
NC5	Lagoon	D0	L4	Derby	STR TET
NC4	Soil	D14	18	Senftenberg	FIS STR TET
NC4	Soil	D21	5	Mbandaka	AMP STR TET
NC4	Soil	D7	1	6,7:-:e,n,z15	FIS STR TET
NC1	Lagoon	D0	L7	Uganda	Pan-susceptible
NC1	Lagoon	D0	L9	Uganda	Pan-susceptible
NC1	Soil	D0	A6	Uganda	AMP AUG AXO FOX FIS GEN STR XNL
NC1	Soil	D7	17	Uganda	AMP AUG AXO FOX FIS GEN STR XNL
NC1	Soil	D0	A20	Worthington	FIS GEN KAN STR TET
NC1	Soil	D7	7	Worthington	FIS GEN KAN STR TET
NC1	Soil	D0	A4	Worthington	FIS GEN KAN STR TET
NC1	Soil	D0	A5	Worthington	FIS GEN KAN STR TET
NC1	Soil	D0	A9	Worthington	FIS GEN KAN STR TET
NC1	Soil	D0	A10	Worthington	FIS GEN KAN STR SXT TET
NC1	Soil	D0	A11	Worthington	FIS GEN KAN STR TET
NC1	Soil	D0	A12	Worthington	FIS GEN KAN STR TET
NC1	Soil	D0	A13	Worthington	FIS GEN KAN STR TET
NC1	Lagoon	D0	L4	Worthington	FIS GEN KAN STR TET
NC3	Soil	D0	A12	Worthington	STR TET
NC3	Soil	D0	A13	Worthington	STR TET
NC3	Soil	D0	A5	Worthington	STR TET
NC3	Soil	D0	A8	Worthington	STR TET
NC3	Soil	D0	A4	Worthington	STR TET
NC5	Soil	D0	A7	Ohio	AMP AUG2 FOX FIS KAN STR TET
NC5	Soil	D7	9	Ouakam	FIS STR TET
IA6	Soil	D0	A14	Litchfield	STR
IA6	Soil	D0	A16	Litchfield	STR
IA6	Soil	D0	A8	Litchfield	STR SXT TET
IA6	Soil	D7	10	Litchfield	STR TET
IA6	Soil	D0	A13	Litchfield	STR
NC3	Soil	D7	1	Typhimurium var5-	FIS KAN STR
NC3	Soil	D7	2	Typhimurium var5-	FIS KAN STR
NC3	Soil	D7	4	Typhimurium var5-	FIS KAN STR TET
NC3	Soil	D7	5	Typhimurium var5-	FIS KAN STR TET
NC3	Soil	D7	10	Typhimurium var5-	AMP FIS KAN STR
NC3	Soil	D7	11	Typhimurium var5-	AMP FIS KAN STR
NC3	Soil	D7	12	Typhimurium var5-	AMP FIS KAN STR
NC3	Soil	D7	13	Typhimurium var5-	AMP FIS KAN STR

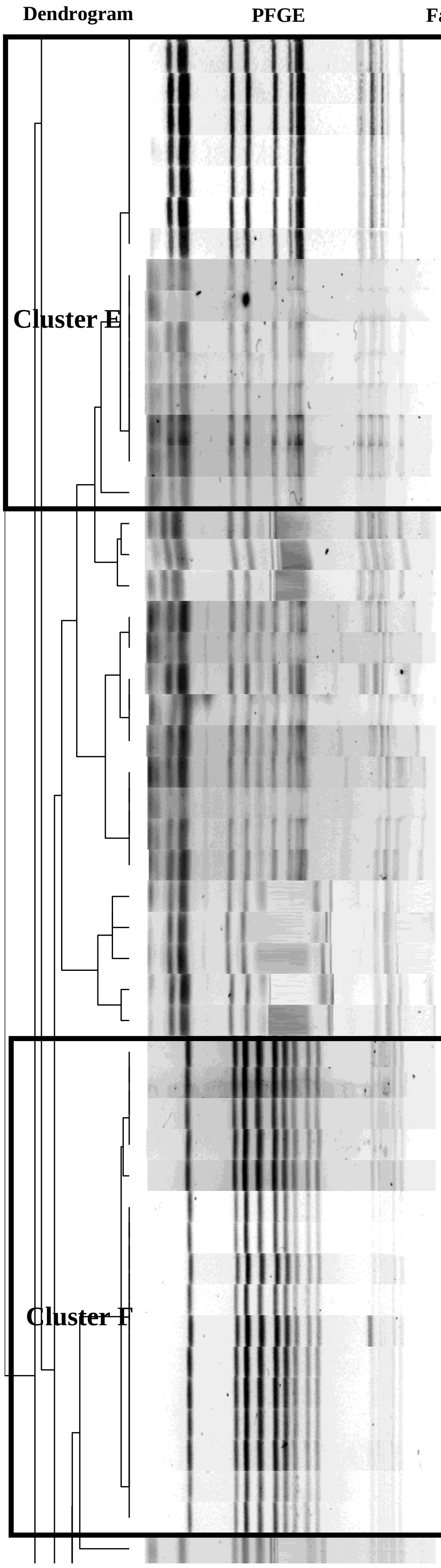

Farms	Sources	Day	ID	Serotypes	R-patterns
NC3	Soil	D7	14	Typhimurium var5-	AMP FIS KAN STR
NC3	Soil	D7	15	Typhimurium var5-	FIS KAN STR
NC3	Soil	D7	16	Typhimurium var5-	AMP FIS KAN STR
NC3	Soil	D7	18	Typhimurium var5-	FIS KAN STR
NC3	Soil	D7	19	Typhimurium var5-	AMP AUG2 FIS KAN STR
NC3	Soil	D7	20	Typhimurium var5-	FIS KAN STR
NC3	Soil	D7	9	Typhimurium var5-	AMP FIS KAN STR
NC3	Soil	D21	12	Typhimurium var5-	FIS KAN STR
NC3	Soil	D21	15	Typhimurium var5-	FIS KAN STR
NC3	Soil	D21	17	Typhimurium var5-	FIS KAN STR
NC3	Soil	D21	2	Typhimurium var5-	FIS KAN STR
NC3	Soil	D21	4	Typhimurium var5-	FIS KAN STR
NC3	Soil	D21	6	Typhimurium var5-	FIS KAN STR
NC3	Soil	D21	7	Typhimurium var5-	FIS KAN STR
NC3	Soil	D21	3	Typhimurium var5-	FIS KAN STR
NC3	Lagoon	D0	L8	Typhimurium var 5-	AMP FIS KAN STR
NC3	Lagoon	D0	L4	Typhimurium var5-	AMP FIS KAN STR
NC3	Soil	D14	19	Typhimurium var5-	AMP FIS KAN STR
NC3	Soil	D14	2	Typhimurium var5-	FIS KAN STR
NC3	Soil	D14	4	Typhimurium var5-	AMP FIS KAN STR
NC3	Soil	D14	1	Typhimurium var5-	FIS KAN STR
NC3	Soil	D14	11	Typhimurium var5-	AMP FIS STR
NC3	Soil	D14	3	Typhimurium var5-	FIS KAN STR
NC3	Soil	D14	6	Typhimurium var5-	AMP FIS KAN STR
NC3	Soil	D14	7	Typhimurium var5-	AMP AUG2 FIS KAN STR
NC3	Soil	D14	8	Typhimurium var5-	AMP FIS KAN STR
NC3	Soil	D14	9	Typhimurium var5-	FIS KAN STR
NC3	Soil	D14	14	Typhimurium var5-	AMP FIS KAN STR
NC3	Soil	D14	16	Typhimurium var5-	AMP FIS KAN STR
NC3	Soil	D14	15	Typhimurium var5-	AMP FIS KAN STR
NC3	Soil	D14	12	Typhimurium var5-	AMP FIS KAN STR
NC3	Soil	D14	13	Typhimurium var5-	AMP FIS KAN STR
NC6	Soil	D0	A13	Rissen	STR TET
NC6	Soil	D0	A18	Rissen	STR TET
NC6	Soil	D0	A20	Rissen	STR TET
NC6	Soil	D0	A7	Rissen	STR TET
NC6	Soil	D0	A19	Rissen	STR TET
NC6	Soil	D0	A2	Rissen	STR TET
NC6	Soil	D0	A4	Rissen	AMP STR TET
NC6	Lagoon	D0	L1	Rissen	STR TET
NC6	Lagoon	D0	L10	Rissen	STR TET
NC6	Lagoon	D0	L3	Rissen	STR TET
NC6	Lagoon	D0	L4	Rissen	STR TET
NC6	Lagoon	D0	L5	Rissen	STR TET
NC6	Lagoon	D0	L6	Rissen	STR TET
NC6	Lagoon	D0	L7	Rissen	STR TET
NC6	Lagoon	D0	L8	Rissen	AMP CHL FIS STR SXT TET
NC6	Lagoon	D0	L9	Rissen	STR TET
NC3	Lagoon	D0	L5	Rissen	STR TET

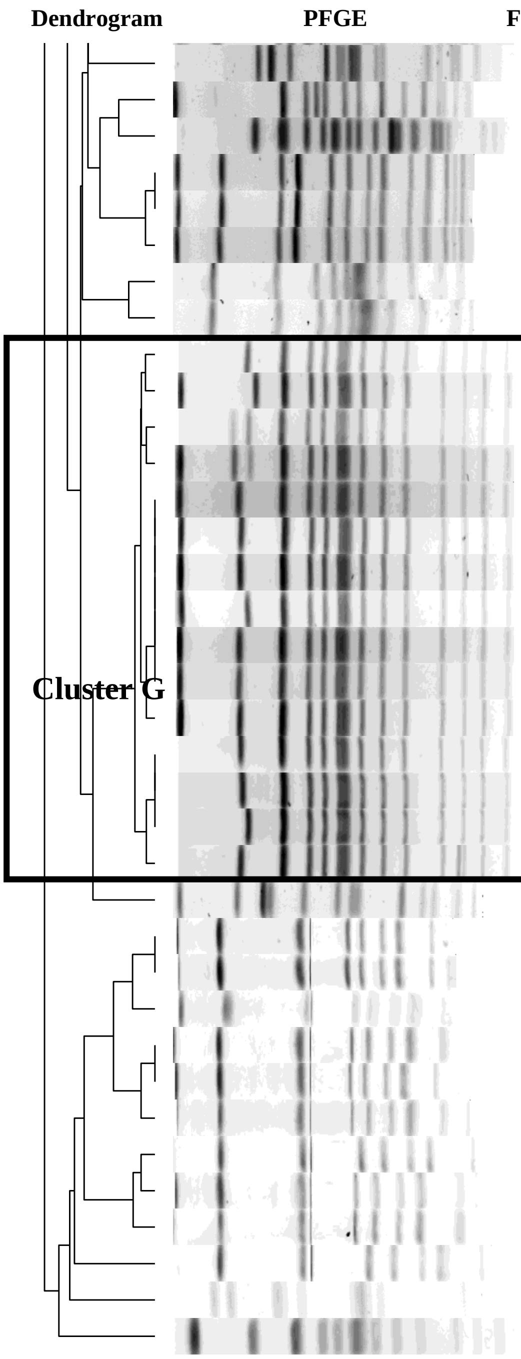

Farms	Sources	Day	ID	Serotypes	R-patterns
NC6	Soil	D0	A8	4,12:i:-	AMP FIS KAN STR TET
IA6	Soil	D0	A18	Infantis	STR
NC1	Soil	D0	A3	Altona	AMP FOX
IA6	Soil	D0	A10	Anatum	FIS STR
IA6	Soil	D7	19	Anatum	STR
IA6	Soil	D0	A20	Anatum	STR
NC2	Lagoon	D7	L5	Derby	FIS STR SXT TET
NC2	Lagoon	D7	L10	Derby	FIS STR SXT TET
NC1	Soil	D0	A1	Muenster	FIS GEN KAN STR TET
NC1	Soil	D0	A16	Muenster	FIS GEN KAN STR TET
NC1	Lagoon	D0	L8	Muenster	FIS GEN KAN STR TET
NC1	Soil	D14	3	Muenster	FIS GEN KAN STR TET
NC1	Soil	D0	A7	Muenster	FIS GEN KAN STR TET
NC1	Soil	D0	A14	Muenster	FIS GEN KAN STR TET
NC1	Soil	D0	A18	Muenster	FIS GEN KAN STR TET
NC1	Soil	D7	13	Muenster	FIS GEN KAN STR TET
NC1	Soil	D14	10	Muenster	FIS GEN KAN STR TET
NC1	Soil	D14	20	Muenster	FIS GEN KAN STR TET
NC1	Soil	D7	5	Muenster	FIS GEN KAN STR TET
NC1	Lagoon	D0	L1	Muenster	FIS GEN KAN STR TET
NC1	Lagoon	D0	L3	Muenster	FIS GEN KAN STR TET
NC1	Lagoon	D0	L5	Muenster	FIS GEN KAN STR TET
NC1	Lagoon	D0	L6	Meunster	FIS GEN KAN STR TET
NC2	Lagoon	D7	L1	Rough_O:z10:e,n,z15	FIS STR SXT TET
IA6	Pit	D0	L6	Anatum	AUG AXO FOX KAN STR XNL
IA6	Pit	D0	L7	Anatum	AMP AUG AXO FOX KAN STR XNL
IA6	Pit	D0	L8	Anatum	AMP AUG AXO FOX KAN STR XNL
IA6	Pit	D0	L4	Anatum	FIS STR
IA6	Pit	D0	L5	Anatum	STR
IA6	Pit	D0	L9	Anatum	AMP AXO FIS STR XNL
IA6	Pit	D0	L2	Anatum	Pan-susceptible
IA6	Pit	D0	L10	Anatum	AXO FOX XNL
IA6	Pit	D0	L3	Anatum	Pan-susceptible
IA6	Pit	D0	L1	Anatum	Pan-susceptible
NC5	Lagoon	D0	L3	Typhimurium var5-	AMP CHL FIS KAN STR TET
NC1	Soil	D7	9	Muenster	FIS GEN KAN STR TET

S1 Fig. Phylogenetic analysis representing PFGE-*Xba*I with antimicrobial resistant patterns of *Salmonella* isolated from NC and IA commercial swine farms at 90% cut-off genotypic similarity (cluster A-G)
